# Ultrasound intima-media thickness cut-off values for the diagnosis of giant cell arteritis using a dual clinical and MRI reference standard and cardiovascular risk stratification

**DOI:** 10.3389/fmed.2024.1389655

**Published:** 2024-04-09

**Authors:** Pascal Seitz, Fabian Lötscher, Susana Bucher, Lukas Bütikofer, Britta Maurer, Arsany Hakim, Luca Seitz

**Affiliations:** ^1^Department of Rheumatology and Immunology, Inselspital, University Hospital Bern, University of Bern, Bern, Switzerland; ^2^CTU Bern, Department of Clinical Research, University of Bern, Bern, Switzerland; ^3^University Institute of Interventional and Diagnostic Neuroradiology, Inselspital, University Hospital Bern, University of Bern, Bern, Switzerland

**Keywords:** giant cell arteritis, vasculitis, ultrasound, cut-off, intima-media thickness, cardiovascular risk, T1-fatsat-black-blood, vessel wall MRI

## Abstract

**Objectives:**

To derive segmental cut-off values and measures of diagnostic accuracy for the intima-media thickness of compressed temporal artery segments for the diagnosis of giant cell arteritis (GCA) on the patient level. To examine the influence of cardiovascular risk.

**Methods:**

Retrospectively, patients evaluated for GCA with an ultrasound of the temporal arteries and an MRI of the head, including a T1-fatsat-black blood (T1-BB) sequence, were identified and classified based on cardiovascular risk and a dual reference standard of T1-BB on the segmental level and the clinical diagnosis on the patient level. Intima-media thickness of the common superficial temporal artery (CSTA), frontal and parietal branches (FB, PB) were measured by compression technique. Statistically and clinically optimal (specificity of approx. 90% for the patient level) cut-offs were derived. Diagnostic accuracy was evaluated on the patient level.

**Results:**

The population consisted of 144 patients, 74 (51.4%) with and 70 (48.6%) without GCA. The *statistically optimal* cut-offs were 0.86 mm, 0.68 mm and 0.67 mm for the CSTA, the FB and PB, respectively. On the patient level sensitivity and specificity were 86.5 and 81.4%. *Clinically optimal* cut-offs were 1.01 mm, 0.82 mm and 0.69 mm and showed a sensitivity of 79.7% and a specificity of 90.0%. For patients *without* high cardiovascular risk, statistically optimal cut-offs showed a sensitivity of 89.6% and a specificity of 90.5%.

**Conclusion:**

Newly derived ultrasound intima-media thickness cut-offs with a dual reference standard show high diagnostic accuracy on the patient level for the diagnosis of GCA, particularly in patients without high cardiovascular risk.

## Introduction

Giant cell arteritis (GCA) often affects the temporal arteries (TAs) and other superficial cranial arteries (SCAs) (1, 2). Timely confirmation of the diagnosis by either imaging and/or biopsy is recommended, with imaging now playing the main role in many centers (3–6). An ultrasound of the SCAs and axillary arteries is the recommended initial imaging test according to the European Alliance of Associations for Rheumatology (EULAR) recommendations in patients with suspected GCA, which includes patients with polymyalgia rheumatica and possible vasculitis (6–8). For magnetic resonance imaging (MRI) of the SCAs, the EULAR recommendations suggest a post-contrast, high-resolution, fat-suppressed T1-weighted, black-blood sequence (T1-BB) on a 3-Tesla scanner, which has a higher diagnostic accuracy for GCA compared to other MRI sequences (6, 9, 10).

Until recently, the presence of a halo or a compression sign was used exclusively to diagnose GCA with ultrasound (6, 11). Although these qualitative signs harbor good diagnostic accuracies, in recent years the measurement of the intima-media thickness (IMT) has gained momentum for the diagnostic evaluation in suspected GCA. The halo and compression signs were defined consensus-based on data using mainly 15 to 18 MHz probes (11, 12). With modern 20 to 24 MHz probes, precise measurements of IMTs are now possible (13–15). The use of diagnostic IMT cut-offs has not yet been adopted widely and is not yet part of the 2023 EULAR recommendations for large vessel vasculitis imaging. Nevertheless, IMT measurements are the basis of the provisional OMERACT ultrasonography score for follow-up in GCA (16).

On the *level of the patient*, GCA can either be present or not. It does not matter whether only a single arterial segment or several arteries are affected. On the *level of the arterial segment,* each segment can possibly be diseased or not. Previous studies about diagnostic IMT cut-offs for the TAs in suspected GCA have used different patient populations and measurement methods (15, 17–19). In these studies, the cut-off values for each segment were derived with the clinical diagnosis of GCA as the diagnostic reference standard on the patient level. Since false positive findings from each individual segment will be added together, a lower specificity is to be expected on the patient level than if each segment is separately evaluated against the diagnostic reference. The situation is different with cranial T1-BB MRI, where the scoring method and the resulting diagnostic accuracy is based on the examination of all available segments together without individual segmental cut-offs (20–22). Ideally, a diagnostic reference should be available for each of the assessed arterial segments. A study with biopsies of each segment is not ethically feasible and would likely yield lower quality data because of the skippy manifestations of GCA. However, by using a double reference standard with the clinical diagnosis on the *patient level* and T1-BB grading on the *segmental level*, this becomes possible.

Using this approach, IMTs for SCAs can be assessed at the *segmental level* using segment-specific cut-off values, with diagnostic accuracy assessment at the *patient level*. Accordingly, at least one segment with an IMT above the segment-specific cut-off is sufficient for a GCA diagnosis. To make ultrasound and MRI examinations comparable, similar lengths of the arteries need to be examined. Most prior ultrasound studies performed IMT measurements only at one specific point or quite focally (15, 17–19). The IMTs were measured either without compression on a single arterial wall or including both walls in a compressed artery, which is the method requiring less time (15, 17–19). Since the examination of a large proportion of the total length of a SCAs is very time-consuming, measuring the IMT in a compressed artery seems more suitable (17).

In daily practice ultrasound is mostly used for ruling *in* the diagnosis of GCA (6). Ruling *out* GCA with an ultrasound of the TAs is not advisable in many cases due to multiple other vessels being possibly affected, e.g., the aorta. Quite often a combination of tests (multiple imaging modalities and/or SCA biopsy) are necessary to rule out GCA, depending on pre-test probability (6, 23). We therefore hypothesized that, from a clinical point of view, cut-off values with high specificity should be aimed for, while from a purely statistical point of view, sensitivity and specificity are usually maximized together for the derivation of optimal cut-off values (24).

Prior studies have shown higher IMT levels in patients with atherosclerosis, with at least one TA segment above published cut-off values in approximately 10–20% of patients at high to very-high cardiovascular risk (CVR) (according to the European Society of Cardiology (ESC) 2021 classification) (25–28). Higher halo scores have also been described in non-GCA patients with high to very-high CVR (28, 29). CVR is therefore expected to influence the diagnostic performance of IMT cut-off values, which is particularly relevant because high CVR is very prevalent in the age group of patients with GCA (28, 30).

The main objective of this study was to derive new segmental cut-off values for the IMT of compressed temporal artery segments [common superficial temporal artery (CSTA), frontal and parietal branches (FB, PB)] with a dual reference standard of T1-BB results on the segment level and the clinical GCA diagnosis on the patient level. These are evaluated together as one examination on the patient level – comparable to an MRI examination – with a sub analysis on the influence of CVR.

## Materials and methods

This is a retrospective, monocentric study, conducted in accordance with the Declaration of Helsinki located at the University Hospital Bern, Switzerland, a tertiary referral center for vasculitis. All patients provided written informed consent for their data to be analyzed. The study was approved by the Ethics Committee Bern, Switzerland, in 2021 (2021–02169). The manuscript fulfills the “Standards for Reporting of Diagnostic Accuracy Studies” (STARD) guidelines (31).

### Study population

Inclusion criteria: ≥ 50 years of age; evaluation for suspected GCA between January 1st 2018 and December 31st 2021; available results of an MRI scan of the head *and* an ultrasound of the SCAs. Exclusion criteria: no informed consent available; severe imaging artifacts; diagnosis of non-GCA vasculitis; missing T1-BB MRI sequence (vessel wall MRI); interval between MRI and ultrasound >7 days. Patients with no documented general consent were specifically contacted by phone and mail and only included if they signed a study-specific consent. The clinical diagnosis ≥6 months after the initial evaluation was used as the diagnostic reference standard on the patient level. It was determined independently by two consultant rheumatologists (L.S., P.S. or F.L.) based on all available electronic medical records (classification as GCA or non-GCA was identical for both experts). Clinical data and ultrasound results were extracted from the electronic patient records and transferred to a coded REDCap database. If certain IMT measurements were missing or unclear on the written report, the archived ultrasound images were double checked to see if these IMT measurements were available or truly missing.

Patients were classified according to CVR. The exact classification into different CVR categories according to ESC 2021 is quite demanding (32). Calculating reliable SCORE2 or SCORE2-OP scores during an assessment in a GCA fast-track clinic is unrealistic, as reliable values on proteinuria, blood lipids and systolic blood pressure must be available, which are very difficult to obtain during an emergency examination in patients who are frequently in pain and under stress (32). Therefore, a pragmatic approach was chosen for the classification into two different groups, which is feasible in the context of a fast-track outpatient assessment. Patients with established atherosclerotic disease (according to available medical records), diabetes mellitus (unless it was of <10 years duration and without known end organ damage or additional known cardiovascular risk factors), moderate to severe chronic renal insufficiency or known familial hypercholesterolemia were classified directly into the high to very-high CVR category according to the ESC 2021 guidelines (32). For all other “apparently healthy” patients (regarding cardiovascular diseases), CVR estimation using ESC-scores would be necessary for classification. This was the second patient group: patients who were not directly allocated to the high/very high CVR group according to ESC 2021 guidelines (32).

### Ultrasound examination technique and scoring

Two different ultrasound machines were used; Logiq E9 from GE (18 MHz transducer) and Canon Aplio i800 (22 MHz transducer) for 27.8 and 72.2% of patients, respectively. Ultrasound examinations were performed by two vasculitis experts (L.S. for 104 patients; F.L. for 40 patients). L.S. and F.L. have performed >1,000 and > 500 vascular ultrasound examinations. The TAs were examined in a supine position, starting in the pretragal region where the TA rises from deep to the parotid gland to approximately the level of the central frontal hair line, for both the PBs and FBs, including sections covered with scalp hair. Ultrasound settings were at the discretion of the examining physician with an aim at maximal resolution and precision for the measurements. Due to artifacts from scalp hair, the B-Mode frequency sometimes had to be lowered to 19 or 20 MHz for the 22 MHz Canon transducer. Doppler was only used for faster identification of the arteries, IMT measurements were exclusively performed in B-Mode images. The bilateral CSTAs, FBs and PBs of the TA were examined in each patient. The segments were completely compressed, i.e., no pulsations and no flow detectable, on transverse view with multiple measurements taken along the length of the examined segment. In a frozen image, the cursor was positioned at both interfaces between the echogenic adventitia and the echo poor combined intima-media as defined by OMERACT; i.e. both single-sided intima-media complexes are combined by the compression and then measured together (16). Clearly atherosclerotic lesions were excluded from measurements, which comprised particularly sites with obvious calcifications. The thickest combined IMT per segment was recorded for each individual segment, i.e., the IMT of both walls were measured together. No additional single sided IMT measurements were carried out in the longitudinal axis, as this would have been too time-consuming due to the length of the examined segments and the number of measurements.

### MRI acquisition, image evaluation, and rating of arteries

All images were acquired on 3-Tesla scanners (Skyra, Prisma and Vida from Siemens Healthineers, Erlangen, Germany) with 20-or 64-channel phased-array head and neck coils. The post-contrast T1-BB sequence was performed as recommended by EULAR and covered the volume from the hard palate to the vertex (6, 33). The sequence parameters were: 30 slices with slice thickness of 3 mm, TR of 500 ms, TE of 22 ms, acquisition matrix of 1′024 × 768, field of view of 200 × 200 mm, axial resolution 0.195 × 0.260 mm (6, 21). The time-of-flight MR-angiography (TOF-MRA) had a slice thickness of 0.5 mm. Readers were blinded to the reference diagnosis and all clinical information apart from age and sex. The coded MRI scans were scored by L.S. (134 scans) and P.S. (10 scans), both senior rheumatologists and vasculitis imaging experts with 13 and 12 years of work experience. The CSTAs, FBs, and PBs were identified bilaterally with the crosshair on corresponding TOF-MRA images, excluding the possibility of accidental identification of a vein (9). Each arterial segment was rated on T1-BB images according to the rating-scheme by Bley et al. (semiquantitative scoring 0 to 3; scores 2 and 3 considered to represent vasculitis) (21). This was used as the diagnostic reference standard on the segmental level.

### Statistics

Statistical analysis was performed using Stata (version 18.0), figures were made with R (version 4.3.1). Patient characteristics are reported as median with interquartile range (IQR) or as absolute and relative frequencies for continuous and categorical variables, respectively. Comparison for continuous and categorical variables was made using the Mann–Whitney-Wilcoxon and Fisher’s exact tests, respectively. Absolute and relative frequencies with Wilson 95%-confidence interval (CI) were used to report the proportion of correct classifications, sensitivity, and specificity. Likelihood ratios are reported with Katz 95%-CIs. *Statistically* optimal cut-offs for the segmental level were determined using the method by Youden (24). The area under the curve (AUC) of the receiver-operating-characteristic (ROC) is reported with asymptotic DeLong 95%-CIs. For the determination of the *statistically optimal* segmental cut-offs, only the data of the following segments were used: as normal segments, all segments of patients without a reference diagnosis of GCA; for pathological segments, only segments from patients with a clinical reference diagnosis of GCA *and* a pathological T1-BB score of 2 or 3. For the determination of the *clinically optimal* segmental cut-offs, the following procedure was chosen: For each of the three segments (CSTA, FB, PB) separately, cut-off values were determined for each 1%-step between a minimum specificity of 85% and a specificity of 100% on the segmental level (for example: the cut-offs giving a minimum specificity of 95% for the CSTA on the segmental level and the cut-offs giving a minimum specificity of 95% for the FB and PB on the segmental level.) Every one of these 16 sets of three cut-offs (one combination for each 1% step of minimum specificity on the segmental level) were then evaluated on the patient level against the reference diagnosis according to the following rule: If at least one segment with an IMT above the cut-off was present, the patient was considered to have GCA. The *clinically optimal* set of three cut-off values was then selected according to the prespecified specificity of approximately 90% at the patient level. The level of 90% specificity was chosen due to the need of a test with high specificity in clinical practice. The same combined evaluation on the patient level was also done for the *statistically optimal* cut-offs.

## Results

From a total of 223 retrospectively identified consecutive patients, 79 patients were excluded from the analysis (Supplementary Figure S1 shows the patient flow chart). The final total patient population included 144 patients, 74 (51.4%) with GCA and 70 (48.6%) with other diagnoses (Supplementary Table S1), of which 23 (32.9%) had polymyalgia rheumatica. The patients with polymyalgia rheumatica all received at least one additional imaging test to screen for possible large vessel vasculitis (22 (95.7%) received an ultrasound of the arm arteries, 13 (56.5%) an ultrasound of the neck arteries, 19 (82.6%) an MRI of the thorax and abdomen and 4 (17.4%) an FDG-PET-CT). None of the 23 included PMR patients had accompanying large vessel vasculitis. Median age was 71 years, and 85 (59.0%) patients were female. Upon clinical presentation, 117 (81.2%) patients had cranial manifestations and 27 (18.8%) patients had only non-cranial signs or symptoms. Patients with GCA were significantly more likely to experience jaw claudication (40.5 vs. 10.0%, *p* < 0.01), new onset headache (75.7 vs. 55.7%, *p* = 0.014) and had higher CRP-levels (mean 82 vs. 54 mg/L, *p* = 0.020). Median time between symptom onset and imaging was 39 days (IQR 15–79 days) for ultrasound and 38 days (IQR 16–78) for MRI. Median duration of therapy with glucocorticoids before imaging was 0 days. For the total population, the median daily prednisolone-equivalent glucocorticoid dose was 0 mg (IQR 0–15 mg) at the time point of US. For the 51/144 (35.4%) patients with glucocorticoid therapy at the time point of US, the median daily prednisolone-equivalent glucocorticoid dose was 45 mg (IQR 14–82 mg). From 144 patients, 43 (29.9%) had documented established atherosclerotic disease. A total of 54 (37.5%) patients belonged to the high/very-high CVR group. There were no significant differences in cardiovascular risk factors and rate of established atherosclerotic disease between GCA and non-GCA cases. Of the 79 (54.9%) patients with a TA biopsy, 42 (53.2%) had vasculitis, defined by the presence of an inflammatory wall infiltrate on histopathology. Table 1 shows the patients’ characteristics for the total population.

**Table 1:** Patients’ characteristics.

Characteristic^a^	Total (*N* = 144)	No GCA^g^ (*N* = 70)	GCA (*N* = 74)	*p*-value
Age (years)^b^	71 (65–76)	69 (62–76)	72 (67–76)	0.07
Female patients	85 (59.0%)	37 (52.9%)	48 (64.9%)	0.18
2022 – ACR/EULAR criteria fulfilled	72 (50.0%)	n.a.^d^	72 (97.3%)	n.a.
New-onset headache	95 (66.0%)	39 (55.7%)	56 (75.7%)	0.014
Scalp tenderness	45 (31.3%)	19 (27.1%)	26 (35.1%)	0.37
Jaw claudication	37 (25.7%)	7 (10.0%)	30 (40.5%)	<0.001
Vision loss^c^	25 (17.4%)	13 (18.6%)	12 (16.2%)	0.11
PMR symptoms	80 (55.6%)	43 (61.4%)	37 (50.0%)	0.18
CRP (mg/L)^b^	64 (22–126)	54 (5–120)	82 (39–130)	0.020
GC therapy at ultrasound	51 (35.4%)	29 (41.4%)	22 (29.7%)	0.17
Duration GC before ultrasound (days)^b^	0 (0–2)	0 (0–3)	0 (0–1)	0.08
GC therapy at MRI	48 (33.3%)	24 (34.3%)	24 (32.4%)	0.86
Duration GC before MRI (days)^b^	0 (0–3)	0 (0–20)	0 (0–1)	0.027
Established atherosclerotic disease^e^	43 (29.9%)	23 (32.9%)	20 (27.0%)	0.47
Diabetes mellitus	12 (8.3%)	6 (8.6%)	6 (8.1%)	1.00
CKD ≥ Grade 3^f^	9 (6.2%)	6 (8.6%)	3 (4.1%)	0.32
Arterial hypertension	73 (50.7%)	36 (51.4%)	37 (50.0%)	0.87
Hypercholesterolemia	37 (25.7%)	21 (30.0%)	16 (21.6%)	0.26

a n (%) unless stated otherwise.

b median (inter quartile range).

c persistent vision loss (complete or incomplete, unilateral or bilateral).

d classification criteria are not met if vasculitis is not present.

e clinically manifest disease.

f chronic kidney disease with KDIGO grading 3 to 5.

g see Supplementary Table S1 for listing of diagnosis. CKD, chronic kidney disease; CRP, C-reactive protein; GC, glucocorticoids; MRI, magnetic resonance imaging; n.a., not applicable; PMR, polymyalgia rheumatica.

### Segment level

**Figure 1:**
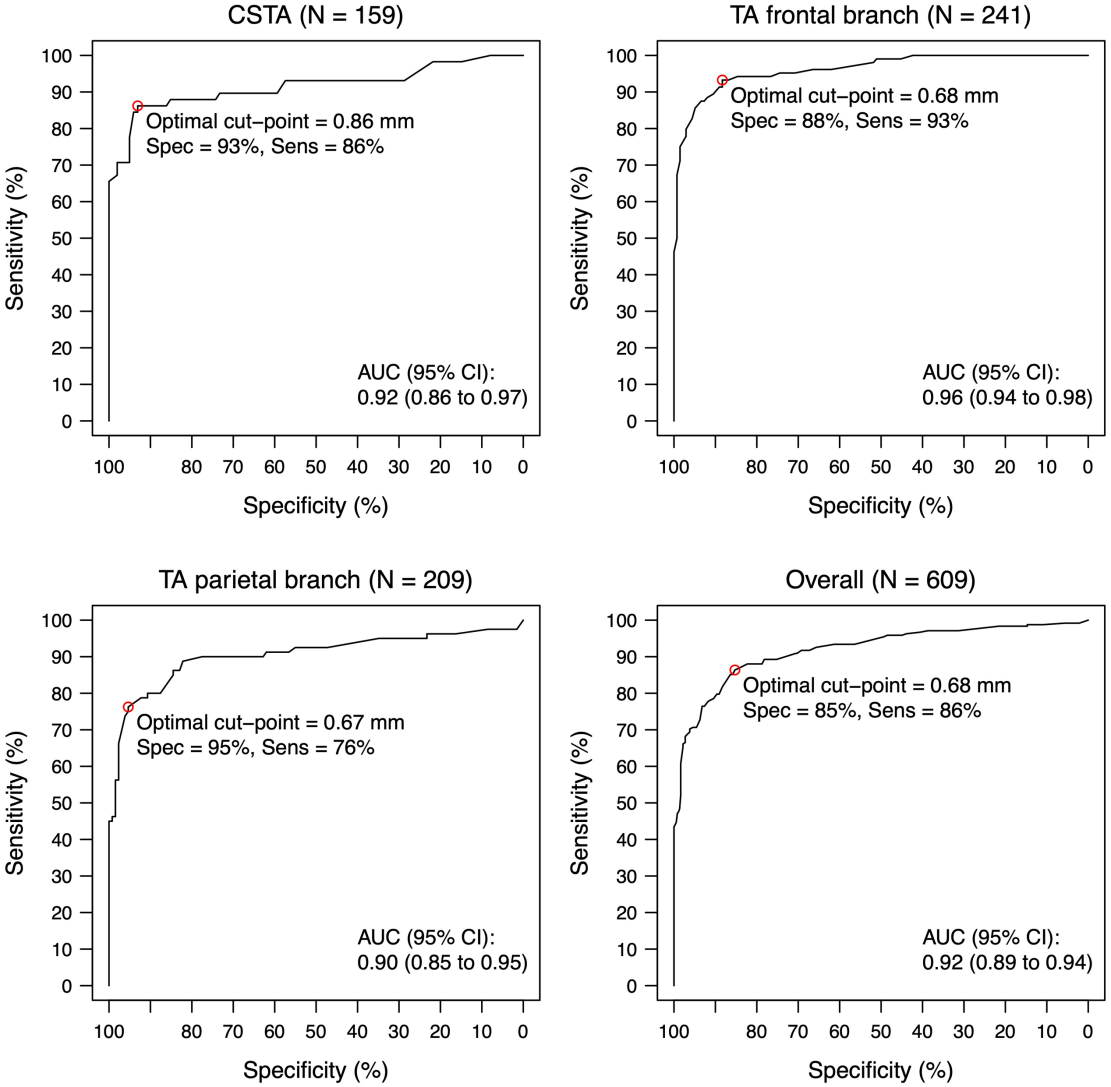
ROC-curve for intima-media thickness for each temporal artery segment and overall. Intima-media thickness values are shown for the compressed lumen technique (combining both walls). The circle indicates the statistically optimal cut-off. Eleven Patients with giant cell arteritis had a normal T1-BB-MRI for all segments and were not included in this analysis. N indicates the number of segments (a maximum of two per patient, left and right side). AUC, area under the curve; CI, confidence interval; CSTA, common superficial temporal artery; IMT, intima-media thickness; ROC, receiver operating characteristic; TA, temporal artery.

Eleven patients with GCA but normal T1-BB-MRI dropped out of the analysis for the derivation of optimal cut-offs. Six of these eleven patients had other pathological test results for vasculitis of the SCA (ultrasound or biopsy); five patients had only extracranial large vessel vasculitis upon imaging. For the total study population, the median IMTs for CSTAs, FBs and PBs were larger for patients with GCA versus without GCA (0.98 mm versus 0.60 mm, 0.91 mm versus 0.48 mm and 0.70 mm versus 0.41 mm, respectively). There were no relevant differences for the IMT between those with and those without cranial symptoms (median IMT for patients with GCA and cranial symptoms: CSTA 1.0 mm, FB 0.99 mm, PB 0.71 mm; no formal testing performed) (Table 2). The *statistically optimal* cut-off for the CSTA was 0.86 mm, with a sensitivity of 86.2% and a specificity of 93.1%; for the FB 0.68 mm, with a sensitivity of 93.3% and a specificity of 88.3%; for the PB 0.67 mm with a sensitivity of 76.2% and a specificity of 95.3%. For all segments together, the *statistically optimal* cut-off was 0.68 mm with a sensitivity of 86.4% and a specificity of 85.3% (Table 3 and Figure 1).

**Table 2:** Intima-media-thickness measurements by ultrasound on segmental level.

Segment	Total (*N* = 288)^a^	No GCA (*N* = 140)^a^	GCA (*N* = 148)^a^	*p*-value
*CSTA*				
N^b^	212	107	105	
Median (IQR)	0.70 (0.55, 1.00)	0.60 (0.50, 0.71)	0.98 (0.70, 1.30)	<0.001
Mean (sd)	0.81 (0.36)	0.61 (0.17)	1.0 (0.39)	
*TA frontal branch*				
N^b^	281	138	143	
Median (IQR)	0.60 (0.45, 0.93)	0.48 (0.40, 0.58)	0.91 (0.66, 1.10)	<0.001
Mean (sd)	0.72 (0.36)	0.50 (0.15)	0.93 (0.37)	
*TA parietal branch*				
N^b^	272	130	142	
Median (IQR)	0.51 (0.40, 0.71)	0.41 (0.35, 0.53)	0.70 (0.49, 0.92)	<0.001
Mean (sd)	0.60 (0.31)	0.44 (0.13)	0.74 (0.35)	

Values for the intima-media thickness are in millimeters, using the compressed lumen technique (combining both walls).

a each patient can have two segments, i.e., the total N is twice the number of patients.

b number of non-missing observations for this segment. CSTA, common superficial temporal artery; GCA, giant cell arteritis; IQR, interquartile range; sd, standard deviation; TA, temporal artery.

**Table 3:** Statistically optimal segment-specific cut-offs.

	Number of patients/segments	Cut-off (mm)	AUC (95% CI)	Sensitivity	Specificity	Positive LR	Negative LR	Correctly classified
CSTA	101/159	0.86	0.92 (0.86–0.97)	86.2% (75.1–92.8%)	93.1% (86.4–96.6%)	12.44 (6.04–25.61)	0.15 (0.08–0.28)	90.6% (85.0–94.2%)
TA frontal branch	129/241	0.68	0.96 (0.94–0.98)	93.3% (86.8–96.7%)	88.3% (81.9–92.7%)	7.99 (5.02–12.69)	0.08 (0.04–0.16)	90.5% (86.1–93.6%)
TA parietal branch	117/209	0.67	0.90 (0.85–0.95)	76.2% (65.9–84.2%)	95.3% (90.2–97.9%)	16.39 (7.43–36.15)	0.25 (0.17–0.37)	88.0% (82.9–91.8%)
Overall^a^	133^b^/609	0.68	0.92 (0.89–0.94)	86.4% (81.5–90.1%)	85.3% (81.3–88.5%)	5.87 (4.57–7.55)	0.16 (0.12–0.22)	85.7% (82.7–88.3%)

Cut-offs for the compressed lumen technique (combining both walls), calculated using method by Youden.

a all segments combined.

b 11 Patients with giant cell arteritis had a normal T1-BB-MRI for all segments and were not included in this analysis. AUC; area under the curve; CSTA, common superficial temporal artery; LR, likelihood ratio; TA, temporal artery.

**Figure 2:**
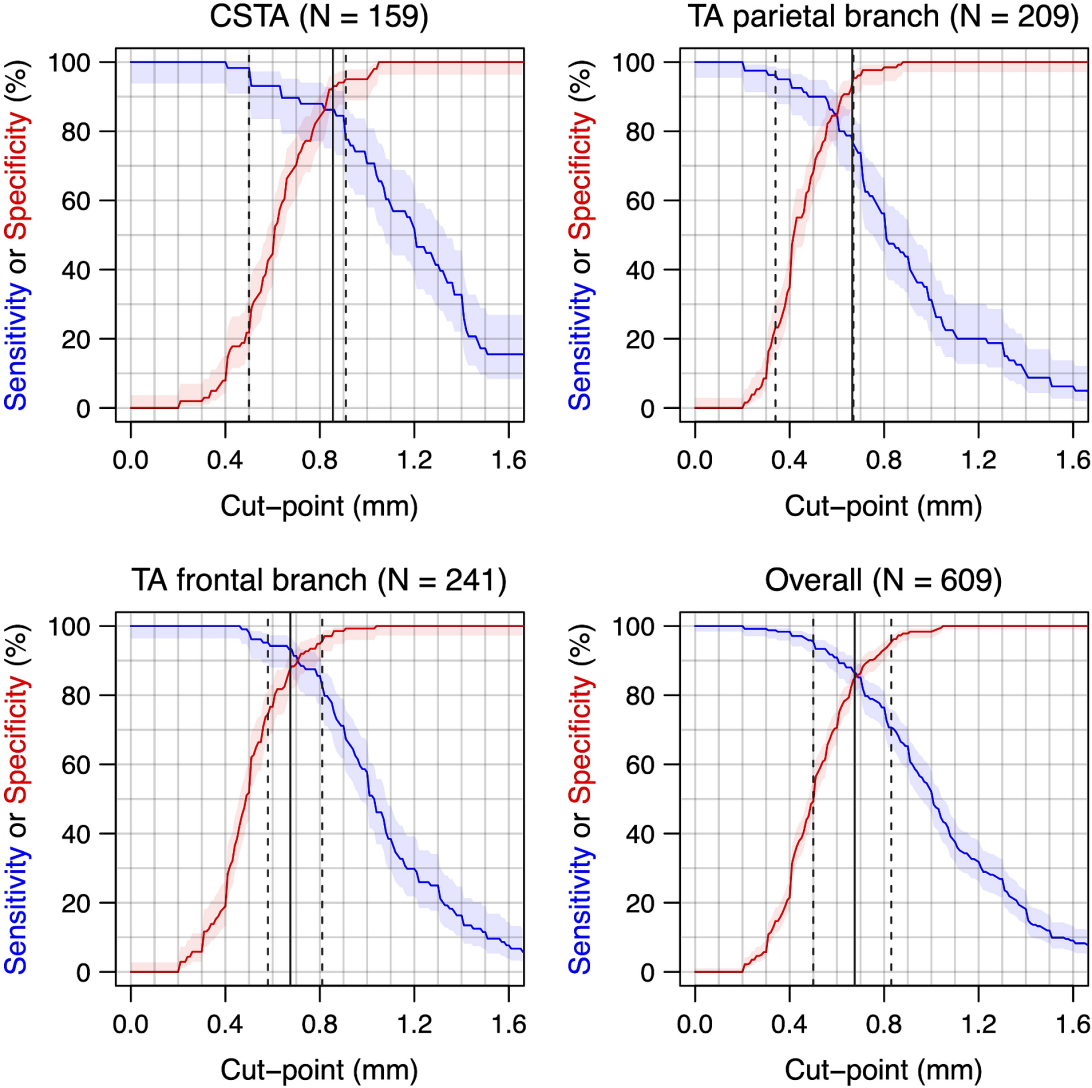
Sensitivity and specificity for different cut-offs for the intima-media thickness for each temporal artery segment and overall. Intima-media thickness values are shown for the compressed lumen technique (combining both walls). Curve for sensitivity: top left to lower right corner. Curve for specificity: lower left to top right corner. Eleven Patients with giant cell arteritis had a normal T1-BB-MRI for all segments and were not included in this analysis. N indicates the number of segments (a maximum of two per patient, left and right side). 95%-confidence regions in shaded areas. The vertical lines indicate the optimal cut-off (solid) and the cut-points to reach a sensitivity or specificity of 95% (dashed). CSTA, common superficial temporal artery; TA, temporal artery.

Figure 2 illustrates the trade-off between sensitivity and specificity depending on the cut-off chosen for each segment and Supplementary Table S2 tabulates these measures of diagnostic accuracy for each 0.1 mm step in the cut-off for each segment and for all segments combined. It can be appreciated that a 0.1 mm step can lead to quite large differences. Table 4 shows the segment-specific cut-offs in millimeters to reach specificities between 85 and 100%: 100% specificity is reached at 1.05 mm, 1.04 mm and 0.88 mm for the CSTA, the FB and the PB, respectively. Supplementary Figure S2 shows positive and negative likelihood ratios depending on the cut-off chosen for each segment. It shows very pronounced likelihood ratios already close to the cut-offs but also the increasing confidence intervals with more extreme cut-offs due to progressively smaller numbers of patients.

**Table 4:** Segment-specific cut-offs to reach specificities of ≥ 85% for all segments.

Minimum specificity for each segment	Common superficial temporal artery	Temporal artery frontal branch	Temporal artery parietal branch
85%	0.81	0.67	0.61
86%	0.82	0.67	0.61
87%	0.83	0.68	0.61
88%	0.83	0.68	0.62
89%	0.83	0.70	0.62
90%	0.84	0.71	0.63
91%	0.84	0.72	0.66
92%	0.84	0.74	0.66
93%	0.86	0.76	0.67
94%	0.88	0.79	0.67
95%	0.91	0.81	0.67
96%	1.01	0.82	0.69
97%	1.02	0.82	0.71
98%	1.03	0.86	0.80
99%	1.05	0.91	0.86
100%	1.05	1.04	0.88

Cut-offs for the compressed lumen technique (combining both walls) are shown in millimeters.

### Patient level

Measures of diagnostic accuracy for the patient level, are shown in Table 5 (a more comprehensive version including source data is included in the supplementary material as Supplementary Table S3). The *statistically optimal* segmental cut-offs, derived from the analysis of individual segments against the clinical reference diagnosis, showed a sensitivity of 86.5% and a specificity of 81.4% for the total study population and a sensitivity of 92.1% and a specificity of 87.0% for the group with cranial manifestations. As expected, the resulting specificities were lower than for the segmental level because results from up to six segments were combined. A specificity of approximately 90% on the patient level was prespecified as criterion for the *clinically optimal* cut-offs. For the total patient population this corresponded to the 96%-specificity cut-off values on the segment level in Table 4. These cut-offs were 1.01 mm for CSTAs, 0.82 mm for FBs and 0.69 mm for PBs. If the ultrasound examination is taken as a whole, at least one of these cut-off values needs to be met to be classified as GCA. These *clinically optimal* cut-off values result in a sensitivity of 79.7% and a specificity of 90.0% for the total study population and a sensitivity of 87.3% and specificity of 94.4% for the group with cranial manifestations. On the patient level the subsets according to CVR were analyzed as well with a clinically relevant advantage in measures of diagnostic accuracies for those patients which did not belong to the high/very-high CVR category. Using the statistically optimal cut-offs for both groups, 40/54 (74.1%) versus 81/90 (90%) were correctly classified; a difference of 15.9% (*p*-value 0.018). For patients with and without high/very-high CVR respectively, sensitivities were 80.8% versus 89.6% and specificities 67.9% versus 90.5%. For clinically optimal cut-offs for the CSTA, the FB and the PB respectively, for patients in the high/very-high CVR group cut-offs were 1.03 mm, 0.86 mm and 0.80 mm with a corresponding sensitivity of 73.1% and a specificity of 89.3%. For patients not in the high/very-high CVR group cut-offs were lower at 0.84 mm, 0.71 mm and 0.63 mm, respectively, with a corresponding sensitivity of 89.6% and specificity of 90.5%. The different sets of cut-off values are shown in Table 6 for easier comparison.

**Table 5:** Patient-level measures of diagnostic accuracy for statistically optimal cut-offs and range of possible cut-offs with *minimum* specificities per segment of 85 to 100%.

	Total study population (*N* = 144)			Patients with cranial manifestations (*N* = 117)			Patients without high/very high CVR (*N* = 90)			Patients with high/very high CVR (*N* = 54)		
	Sensitivity (95% CI)	Specificity (95% CI)	Correctly classified (95% CI)	Sensitivity (95% CI)	Specificity (95% CI)	Correctly classified (95% CI)	Sensitivity (95% CI)	Specificity (95% CI)	Correctly classified (95% CI)	Sensitivity (95% CI)	Specificity (95% CI)	Correctly classified (95% CI)
Statistically optimal cut-offs	86.5% (76.9–92.5%)	81.4% (70.8–88.8%)	84.0% (77.2–89.1%)	92.1% (82.7–96.6%)	87% (75.6–93.6%)	89.7% (82.9–94.0%)	89.6% (77.8–95.5%)	90.5% (77.9–96.2%)	90.0% (82.1–94.5%)	80.8% (62.1–91.5%)	67.9% (49.3–82.1%)	74.1% (61.1–83.9%)
Specificity 85%	86.5% (76.9–92.5%)	75.7% (64.5–84.2%)	81.2% (74.1–86.8%)	92.1% (82.7–96.6%)	79.6% (67.1–88.2%)	86.3% (78.9–91.4%)	89.6% (77.8–95.5%)	85.7% (72.2–93.3%)	87.8% (79.4–93.0%)	80.8% (62.1–91.5%)	60.7% (42.4–76.4%)	70.4% (57.2–80.9%)
Specificity 86%	86.5% (76.9–92.5%)	75.7% (64.5–84.2%)	81.2% (74.1–86.8%)	92.1% (82.7–96.6%)	79.6% (67.1–88.2%)	86.3% (78.9–91.4%)	89.6% (77.8–95.5%)	85.7% (72.2–93.3%)	87.8% (79.4–93.0%)	80.8% (62.1–91.5%)	60.7% (42.4–76.4%)	70.4% (57.2–80.9%)
Specificity 87%	86.5% (76.9–92.5%)	77.1% (66.0–85.4%)	81.9% (74.9–87.4%)	92.1% (82.7–96.6%)	81.5% (69.2–89.6%)	87.2% (79.9–92.1%)	89.6% (77.8–95.5%)	88.1% (75.0–94.8%)	88.9% (80.7–93.9%)	80.8% (62.1–91.5%)	60.7% (42.4–76.4%)	70.4% (57.2–80.9%)
Specificity 88%	86.5% (76.9–92.5%)	77.1% (66.0–85.4%)	81.9% (74.9–87.4%)	92.1% (82.7–96.6%)	81.5% (69.2–89.6%)	87.2% (79.9–92.1%)	89.6% (77.8–95.5%)	88.1% (75.0–94.8%)	88.9% (80.7–93.9%)	80.8% (62.1–91.5%)	60.7% (42.4–76.4%)	70.4% (57.2–80.9%)
Specificity 89%	86.5% (76.9–92.5%)	77.1% (66.0–85.4%)	81.9% (74.9–87.4%)	92.1% (82.7–96.6%)	81.5% (69.2–89.6%)	87.2% (79.9–92.1%)	89.6% (77.8–95.5%)	88.1% (75.0–94.8%)	88.9% (80.7–93.9%)	80.8% (62.1–91.5%)	60.7% (42.4–76.4%)	70.4% (57.2–80.9%)
Specificity 90%	86.5% (76.9–92.5%)	78.6% (67.6–86.6%)	82.6% (75.6–88.0%)	92.1% (82.7–96.6%)	81.5% (69.2–89.6%)	87.2% (79.9–92.1%)	89.6% (77.8–95.5%)	90.5% (77.9–96.2%)	90.0% (82.1–94.6%)	80.8% (62.1–91.5%)	60.7% (42.4–76.4%)	70.4% (57.2–80.9%)
Specificity 91%	86.5% (76.9–92.5%)	80.0% (69.2–87.7%)	83.3% (76.4–88.5%)	92.1% (82.7–96.6%)	83.3% (71.3–91.0%)	88.0% (80.9–92.7%)	89.6% (77.8–95.5%)	92.9% (81.0–97.5%)	91.1% (83.4–95.4%)	80.8% (62.1–91.5%)	60.7% (42.4–76.4%)	70.4% (57.2–80.9%)
Specificity 92%	85.1% (75.3–91.5%)	80.0% (69.2–87.7%)	82.6% (75.6–88.0%)	92.1% (82.7–96.6%)	83.3% (71.3–91.0%)	88.0% (80.9–92.7%)	87.5% (75.3–94.1%)	92.9% (81.0–97.5%)	90.0% (82.1–94.6%)	80.8% (62.1–91.5%)	60.7% (42.4–76.4%)	70.4% (57.2–80.9%)
Specificity 93%	85.1% (75.3–91.5%)	85.7% (75.7–92.1%)	85.4% (78.7–90.3%)	92.1% (82.7–96.6%)	90.7% (80.1–96.0%)	91.5% (85.0–95.3%)	87.5% (75.3–94.1%)	95.2% (84.2–98.7%)	91.1% (83.4–95.4%)	80.8% (62.1–91.5%)	71.4% (52.9–84.7%)	75.9% (63.1–85.4%)
Specificity 94%	83.8% (73.8–90.5%)	85.7% (75.7–92.1%)	84.7% (78.0–89.7%)	92.1% (82.7–96.6%)	90.7% (80.1–96.0%)	91.5% (85.0–95.3%)	87.5% (75.3–94.1%)	95.2% (84.2–98.7%)	91.1% (83.4–95.4%)	76.9% (57.9–89.0%)	71.4% (52.9–84.7%)	74.1% (61.1–83.9%)
Specificity 95%	81.1% (70.7–88.4%)	85.7% (75.7–92.1%)	83.3% (76.4–88.5%)	88.9% (78.8–94.5%)	90.7% (80.1–96.0%)	89.7% (82.9–94.0%)	83.3% (70.4–91.3%)	95.2% (84.2–98.7%)	88.9% (80.7–93.9%)	76.9% (57.9–89.0%)	71.4% (52.9–84.7%)	74.1% (61.1–83.9%)
Specificity 96%	79.7% (69.2–87.3%)	90.0% (80.8–95.1%)	84.7% (78.0–89.7%)	87.3% (76.9–93.4%)	94.4% (84.9–98.1%)	90.6% (83.9–94.7%)	83.3% (70.4–91.3%)	97.6% (87.7–99.6%)	90.0% (82.1–94.6%)	73.1% (53.9–86.3%)	78.6% (60.5–89.8%)	75.9% (63.1–85.4%)
Specificity 97%	77.0% (66.3–85.1%)	91.4% (82.5–96.0%)	84.0% (77.2–89.1%)	84.1% (73.2–91.1%)	94.4% (84.9–98.1%)	88.9% (81.9–93.4%)	79.2% (65.7–88.3%)	97.6% (87.7–99.6%)	87.8% (79.4–93.0%)	73.1% (53.9–86.3%)	82.1% (64.4–92.1%)	77.8% (65.1–86.8%)
Specificity 98%	73.0% (61.9–81.8%)	94.3% (86.2–97.8%)	83.3% (76.4–88.5%)	79.4% (67.8–87.5%)	96.3% (87.5–99.0%)	87.2% (79.9–92.1%)	72.9% (59.0–83.4%)	97.6% (87.7–99.6%)	84.4% (75.6–90.5%)	73.1% (53.9–86.3%)	89.3% (72.8–96.3%)	81.5% (69.2–89.6%)
Specificity 99%	68.9% (57.7–78.3%)	98.6% (92.3–99.7%)	83.3% (76.4–88.5%)	74.6% (62.7–83.7%)	98.1% (90.2–99.7%)	85.5% (78.0–90.7%)	68.8% (54.7–80.1%)	100% (91.6–100%)	83.3% (74.3–89.6%)	69.2% (50.0–83.5%)	96.4% (82.3–99.4%)	83.3% (71.3–91.0%)
Specificity 100%	63.5% (52.1–73.6%)	100% (94.8–100%)	81.2% (74.1–86.8%)	68.3% (56.0–78.4%)	100% (93.4–100%)	82.9% (75.1–88.7%)	62.5% (48.4–74.8%)	100% (91.6–100%)	80.0% (70.6–87.0%)	65.4% (46.2–80.6%)	100% (87.9–100%)	83.3% (71.3–91.0%)

GCA, giant cell arteritis; CI, confidence interval; CVR, cardiovascular risk; N, number of patients in the subpopulation.

**Table 6:** Comparison of statistically optimal and clinically optimal cut-offs with associated sensitivities and specificities on the patient level.

	Statistically optimal cut-offs total study population	Clinically optimal^a^ cut-offs total study population	Clinically optimal^a^ cut-offs with high/very high CVR	Clinically optimal^a^ cut-offs without high/very CVR
Common superficial TA	0.86 mm	1.01 mm	1.03 mm	0.84 mm
Frontal branch of TA	0.68 mm	0.82 mm	0.86 mm	0.71 mm
Parietal branch of TA	0.67 mm	0.69 mm	0.80 mm	0.63 mm
Sensitivity	86.5% (76.9–92.5%)	79.7% (69.2–87.3%)	73.1% (53.9–86.3%)	89.6% (77.8–95.5%)
Specificity	81.4% (70.8–88.8%)	90.0% (80.8–95.1%)	89.3% (72.8–96.3%)	90.5% (77.9–96.2%)

Cut-off values are shown for the compressed lumen technique (combining both walls).

a defined as sets of cut-offs with approximately 90% specificity on the patient level (from Tables 4, 5). CVR, cardiovascular risk; TA, temporal artery.

## Discussion

This study evaluated patients with suspected GCA, *including isolated non-cranial presentations*, in a real-life scenario. Segmental cut-off values for the IMT of the TA segments were evaluated for the diagnosis of GCA at the patient level, comparable to the usual approach of cranial MRI. An innovative and novel approach with a double reference standard of expert clinical diagnosis at the patient level and T1-BB-MRI results at the segment level was used to identify normal and diseased segments for the derivation of new cut-off values.

In daily clinical practice, *ruling in* GCA is the focus of imaging studies as *ruling out* GCA with an ultrasound examination of the TAs is often not possible (6, 34). While in the case of a negative test, another test is usually performed depending on pre-test probability for GCA, the use of a test with high specificity is important to limit false-positive results. Therefore, in addition to *statistically optimal* cut-off values, we derived *clinically optimal* cut-off values with a predefined specificity of approximately 90% or higher on the patient level, which allows a GCA diagnosis with a high degree of certainty, particularly in patients with a high pretest probability.

The *statistically optimal* segmental cut-offs for the total study population obtained using the compression technique are very similar to previously published cut-offs, which were mostly reported as single-sided measurements (our values would need to be divided by two for direct comparison) (15, 17–19).

The focus of this study was the combined assessment of all segments together on the patient level, which is the relevant test in daily practice. Using a combination of several segments together, a loss in specificity can be expected compared to an analysis with single segments because false positives become more likely. Using the newly derived *statistically optimal* cut-offs from the segment level analysis, a sensitivity and specificity of 86.7%/81.4 and 92.1%/87.0% was reached for the patient level for the total population and patients with cranial manifestations, respectively. The use of *clinically optimal* segmental cut-off values was associated with a slight drop in sensitivity to 79.7% at a specificity of 90.0% for the total study population. For patients with cranial manifestations, the diagnostic accuracy was higher and the drop in sensitivity to 87.3% less pronounced. The *clinically optimal* cut-offs are 0.02 to 0.15 mm higher than the *statistically optimal* cut-offs (compressed artery) (Table 6).

The mean IMT shows considerably lower values for the frontal and especially the parietal branches in both non-GCA and GCA cases compared to the CSTA (Table 2). This justifies the use of segment-specific cut-off values for the diagnosis of GCA.

Our cohort had a considerable proportion of patients with high/very-high CVR and subgroup analysis showed pronounced differences in measures of diagnostic accuracy. The 22.6% lower specificity for newly derived *statistically optimal* cut-offs for patients with high/very-high CVR is striking but corresponds well to our clinical experience. In other words, in order to achieve a specificity of around 90% for the patient level in individuals with high/very-high CVR, the cut-offs need to be raised considerably. However, for patients without high/very-high CVR, *clinically optimal* cut-offs are much lower and correspond approximately to the *statistically optimal* cut-offs for the total study population (Table 6 for direct comparison of different sets of cut-off values).

Using the data from our study, ultrasound results can be used in a Bayesian approach to the diagnosis of GCA, depending on clinical circumstances. Using *clinically optimal* cut-offs would allow to *rule in* a GCA diagnosis in cases with reasonably high pre-test probability but with some compromises in sensitivity. In the case of a negative ultrasound, other diagnostic tests such as cranial MRI, TA biopsy or FDG-PET-CT can be performed, and we have been using this stepwise approach successfully in clinical practice for several years (6). The measures of diagnostic accuracy from our study compare well to recent pooled estimates for the T1-BB MRI (sensitivity of 82%, specificity of 92%) (6, 35). Since a relevant proportion of patients with GCA do not have vasculitis of the TAs, the maximum attainable sensitivity of any diagnostic test for the TAs is expected to lie below 100% with an expected ceiling effect (34, 36).

When the results of this study are compared with previously published data, some important differences need to be considered. Comparison of this study to the only other study using IMT measurements in the compressed artery by Czihal et al. is complicated by the fact that they did not differentiate TA segments (17). Mean IMT for GCA patients was reported as 1.03 mm with a standard deviation (SD) of only 0.03 mm; for non-GCA cases it was 0.44 mm (SD 0.13 mm). Values for mean IMT are in line with our results but the SD for GCA cases is much smaller than in our cohort where SDs were more than ten times larger for GCA cases (see Table 2). Czihal et al. (17) One possible explanation of higher variability in IMT values could be that in our study multiple measurements were taken along a large section of the artery, compared to mostly defined single point or more limited measurements in previous studies, also in the study by Czihal et al. (15, 17–19). In addition, areas with scalp hair, where measurements can be challenging, were also included. Schäfer et al. used a very different patient population and selection procedure of relevant arterial segments, making a direct comparison to the present study difficult. They published the first estimates for cut-off values in 2017, which are similar to the *statistically optimal* cut-offs (divided by two) for the total study population from the present study, with only the PB having a relevantly lower value (15). Newer studies using single-sided measurements published very similar segmental cut-off values. Ješe et al. (18) used a comparable patient population but used a probe with non-adjustable 18 MHz and single-sided longitudinal IMT measurements (18). Despite generating an overall cut-off of 0.40 mm for all TA-segments combined, very high estimates for sensitivity and specificity were presented (97.9 and 99.0%) (18). The study by López-Gloria et al. from 2022 also used a similar population, an 18 MHz probe with longitudinal single-sided IMT measurements with focal measurements 1 cm distal to the TA bifurcation in the PB and FB and derived similar segmental cut-off values but with very high sensitivities and specificities (94.7–100%) (19). Measures of diagnostic accuracies for segmental and patient level (the latter only by Ješe et al.) analysis from these studies surpassed those from MRI studies and our data considerably (18, 19, 35). While the more comprehensive IMT measurement method in the present study is a likely explanatory factor, there may be unknown differences in study design or patient population as well. Furthermore, the exact handling of measurements at locations with possible atherosclerotic disease may have been a relevant source of heterogeneity between studies. OMERACT provides a definition of atherosclerotic vessel wall changes with an emphasis on echogenicity (11). Despite that, the clear differentiation of atherosclerosis and/or intima hyperplasia from vasculitis with ultrasound remains extremely challenging, especially in cases where atherosclerosis and vasculitis coexist, which is frequent in patients with high CVR. We believe it is particularly in patients with atherosclerosis, where it is the most difficult to differentiate diseased from non-diseased segments. The drop in specificity in the subgroup analysis with high/very-high CVR demonstrates this clearly.

This study has several limitations. Patients were retrospectively collected but represent a typical population from a tertiary referral center for suspected GCA. The combined IMT of both walls of a compressed artery is measured at our center because multiple measurements would be very time consuming with the single-sided longitudinal method and becomes even more difficult and sometimes impossible in areas with scalp hair. A direct comparison of IMT measurements of compressed arteries with single-sided IMT measurements seems reasonable, and OMERACT regards this method as equivalent, but, to our knowledge, it has not been proven that both methods result in equal results (16). Single-sided measurements with their shorter distances place considerably higher demands on the ultrasound equipment and may therefore be less widely applicable internationally. Both, ultrasound examinations and re-reading of MRI images, were not done by 2 independent readers because of the retrospective nature of the data for the former and time constraints for the latter. Therefore, no information on inter-rater reliability can be provided for the ultrasound measurements. For the T1-BB MRI, an inter-rater analysis for the same two readers was published previously for another study and showed substantial reliability (9). The pragmatic classification into two CVR groups is possibly imperfect, as some of the patients may be re-classified into the high/very-high CVR group after application of the SCORE2/SCORE2-OP-scores, especially elderly men in high-or very-high-risk countries (e.g., Eastern Europe; Switzerland is a low-risk country) (32). While a perfect classification into CVR groups would be ideal, in our opinion this is not feasible in the situation of a fast-track clinic. Even in the inpatient setting, the application of a SCORE-score is difficult because blood pressure measurements in patients with pain and high dose glucocorticoids are not reliable. The method proposed in this study allows a pragmatic classification at the bedside using readily available clinical information. The cut-offs were derived from and evaluated in the same population. Still, for the derivation of measures of diagnostic accuracy on the patient level all 765 available segments were used, while for the cut-off derivation only 609 segments were used. A formal prospective validation on another and eventually also external patient population is necessary.

Because of the segmental manifestation of the disease and the potential influence of sex, height, weight, age and CVR on IMT, the diagnostic approach using IMT cut-off values remains complex and still has its limitations (37). An even better conceptualization of the diagnostic process for GCA, including pertinent features from the patient history, the physical examination and laboratory values would be a multivariable model including IMTs as continuous variables. Including information on CVR would allow an estimation of the influence of the IMT on the probability of disease independent of this risk categorization. In such models, MRI data could be implemented as well. For adequate derivation of such multivariate models, larger number of patients in a prospective study design may be necessary, depending on the number of variables used.

In conclusion, our study provides four sets of segmental cut-offs with measures of diagnostic accuracy for the diagnosis of giant cell arteritis for direct application in clinical practice depending on the clinical situation and physician preference.

## Data availability statement

The raw data supporting the conclusions of this article will be made available by the authors, without undue reservation.

## Ethics statement

The studies involving humans were approved by Ethics Committee Bern, Switzerland. The studies were conducted in accordance with the local legislation and institutional requirements. The participants provided their written informed consent to participate in this study.

## Author contributions

PS: Conceptualization, Data curation, Formal analysis, Funding acquisition, Investigation, Methodology, Project administration, Resources, Visualization, Writing – original draft, Writing – review & editing. FL: Conceptualization, Data curation, Investigation, Methodology, Resources, Writing – review & editing. SB: Data curation, Investigation, Writing – review & editing. LB: Formal analysis, Visualization, Writing – review & editing. BM: Funding acquisition, Resources, Writing – review & editing. AH: Resources, Writing – review & editing. LS: Conceptualization, Data curation, Formal analysis, Funding acquisition, Investigation, Methodology, Project administration, Resources, Supervision, Visualization, Writing – original draft, Writing – review & editing.

## References

[1] Ponte C Martins-Martinho J Luqmani RA. Diagnosis of giant cell arteritis. Rheumatology (Oxford). (2020) 59:iii5–iii16. doi: 10.1093/rheumatology/kez55 32348512

[2] Seitz L Seitz P Pop R Lötscher F. Spectrum of large and medium vessel Vasculitis in adults: primary Vasculitides, Arthritides, connective tissue, and fibroinflammatory diseases. Curr Rheumatol Rep. (2022) 24:352–70. doi: 10.1007/s11926-022-01086-2 36166150 PMC9513304

[3] Vodopivec I Rizzo JF 3rd. Ophthalmic manifestations of giant cell arteritis. Rheumatology (Oxford). (2018) 57:ii63–72. doi: 10.1093/rheumatology/kex428 29986083

[4] Diamantopoulos AP Haugeberg G Lindland A Myklebust G. The fast-track ultrasound clinic for early diagnosis of giant cell arteritis significantly reduces permanent visual impairment: towards a more effective strategy to improve clinical outcome in giant cell arteritis? Rheumatology (Oxford). (2016) 55:66–70. doi: 10.1093/rheumatology/kev289 26286743

[5] Hellmich B Agueda A Monti S Buttgereit F de Boysson H Brouwer E. 2018 update of the EULAR recommendations for the management of large vessel vasculitis. Ann Rheum Dis. (2020) 79:19–30. doi: 10.1136/annrheumdis-2019-215672 31270110

[6] Dejaco C Ramiro S Bond M Bosch P Ponte C Mackie SL. EULAR recommendations for the use of imaging in large vessel vasculitis in clinical practice. Ann Rheum Dis. (2023) 2023. doi: 10.1136/ard-2023-224543 37550004

[7] Tomelleri A van der Geest KSM Khurshid MA Sebastian A Coath F Robbins D. Disease stratification in GCA and PMR: state of the art and future perspectives. Nat Rev Rheumatol. (2023) 19:446–59. doi: 10.1038/s41584-023-00976-8 37308659

[8] Seitz P Cullmann J Bucher S Bütikofer L Reichenbach S Lötscher F. Musculoskeletal magnetic resonance imaging findings support a common spectrum of giant cell arteritis and polymyalgia rheumatica. Rheumatology. (2024). doi: 10.1093/rheumatology/keae043 38265241

[9] Seitz L Bucher S Bütikofer L Maurer B Bonel HM Wagner F. Diffusion-weighted magnetic resonance imaging for the diagnosis of giant cell arteritis - a comparison with T1-weighted black-blood imaging. Rheumatology. (2023). doi: 10.1093/rheumatology/kead401 37555808

[10] Geiger J Bley T Uhl M Frydrychowicz A Langer M Markl M. Diagnostic value of T2-weighted imaging for the detection of superficial cranial artery inflammation in giant cell arteritis. J Magn Reson Imaging. (2010) 31:470–4. doi: 10.1002/jmri.22047 20099359

[11] Chrysidis S Duftner C Dejaco C Schäfer VS Ramiro S Carrara G. Definitions and reliability assessment of elementary ultrasound lesions in giant cell arteritis: a study from the OMERACT large vessel vasculitis ultrasound working group. RMD Open. (2018) 4:e000598. doi: 10.1136/rmdopen-2017-000598 29862043 PMC5976098

[12] Schmidt WA Kraft HE Vorpahl K Völker L Gromnica-Ihle EJ. Color duplex ultrasonography in the diagnosis of temporal arteritis. N Engl J Med. (1997) 337:1336–42. doi: 10.1056/NEJM199711063371902 9358127

[13] Seitz L Christ L Lötscher F Scholz G Sarbu AC Bütikofer L. Quantitative ultrasound to monitor the vascular response to tocilizumab in giant cell arteritis. Rheumatology (Oxford). (2021) 60:5052–9. doi: 10.1093/rheumatology/keab484 34117737 PMC8566271

[14] Nielsen BD Therkildsen P Keller KK Gormsen LC Hansen IT Hauge EM. Ultrasonography in the assessment of disease activity in cranial and large-vessel giant cell arteritis: a prospective follow-up study. Rheumatology (Oxford). (2023) 62:3084–94. doi: 10.1093/rheumatology/kead028 36651670

[15] Schäfer VS Juche A Ramiro S Krause A Schmidt WA. Ultrasound cut-off values for intima-media thickness of temporal, facial and axillary arteries in giant cell arteritis. Rheumatology. (2017) 56:1632. doi: 10.1093/rheumatology/kex143 28859330

[16] Dejaco C Ponte C Monti S Rozza D Scirè CA Terslev L. The provisional OMERACT ultrasonography score for giant cell arteritis. Ann Rheum Dis. (2023) 82:556–64. doi: 10.1136/ard-2022-223367 36600183

[17] Czihal M Schröttle A Baustel K Lottspeich C Dechant C Treitl KM. B-mode sonography wall thickness assessment of the temporal and axillary arteries for the diagnosis of giant cell arteritis: a cohort study. Clin Exp Rheumatol. (2017) 35:128–33. 28375835

[18] Ješe R Rotar Ž Tomšič M Hočevar A. The cut-off values for the intima-media complex thickness assessed by colour doppler sonography in seven cranial and aortic arch arteries. Rheumatology (Oxford). (2021) 60:1346–52. doi: 10.1093/rheumatology/keaa578 32944770

[19] López-Gloria K Castrejón I Nieto-González JC Rodríguez-Merlos P Serrano-Benavente B González CM. Ultrasound intima media thickness cut-off values for cranial and extracranial arteries in patients with suspected giant cell arteritis. Front Med. (2022) 9:981804. doi: 10.3389/fmed.2022.981804 PMC9459085 36091695

[20] Bley TA Weiben O Uhl M Vaith P Schmidt D Warnatz K. Assessment of the cranial involvement pattern of giant cell arteritis with 3T magnetic resonance imaging. Arthritis Rheum. (2005) 52:2470–7. doi: 10.1002/art.21226 16052572

[21] Bley TA Wieben O Uhl M Thiel J Schmidt D Langer M. High-resolution MRI in giant cell arteritis: imaging of the wall of the superficial temporal artery. AJR Am J Roentgenol. (2005) 184:283–7. doi: 10.2214/ajr.184.1.01840283 15615989

[22] Klink T Geiger J Both M Ness T Heinzelmann S Reinhard M. Giant cell arteritis: diagnostic accuracy of MR imaging of superficial cranial arteries in initial diagnosis-results from a multicenter trial. Radiology. (2014) 273:844–52. doi: 10.1148/radiol.14140056 25102371

[23] Lecler A Hage R Charbonneau F Vignal C Sené T Picard H. Validation of a multimodal algorithm for diagnosing giant cell arteritis with imaging. Diagn Interv Imaging. (2022) 103:103–10. doi: 10.1016/j.diii.2021.09.008 34663548

[24] Youden WJ. Index for rating diagnostic tests. Cancer. (1950) 3:32–5. doi: 10.1002/1097-0142(1950)3:1<32::aid-cncr2820030106>3.0.co;2-3 15405679

[25] Seitz L Lötscher F. The intima-media thickness in suspected giant cell arteritis-sometimes it is worth taking a closer look. Rheumatology. (2021) 60:3039–41. doi: 10.1093/rheumatology/keab316 33774656

[26] De Miguel E Beltran LM Monjo I Deodati F Schmidt WA Garcia-Puig J. Atherosclerosis as a potential pitfall in the diagnosis of giant cell arteritis. Rheumatology (Oxford). (2018) 57:318–21. doi: 10.1093/rheumatology/kex381 29112741

[27] Martire MV Cipolletta E Di Matteo A Di Carlo M Jesus D Grassi W. Is the intima-media thickness of temporal and axillary arteries influenced by cardiovascular risk? Rheumatology. (2021) 60:5362–8. doi: 10.1093/rheumatology/keab117 33547776

[28] Molina-Collada J López Gloria K Castrejón I Nieto-González JC Martínez-Barrio J Anzola Alfaro AM. Impact of cardiovascular risk on the diagnostic accuracy of the ultrasound Halo Score for giant cell arteritis. Arthritis Res Ther. (2022) 24:232. doi: 10.1186/s13075-022-02920-9 36229861 PMC9558391

[29] van der Geest KSM Borg F Kayani A Paap D Gondo P Schmidt W. Novel ultrasonographic halo score for giant cell arteritis: assessment of diagnostic accuracy and association with ocular ischemia. Ann Rheum Dis. (2020) 79:393–9. doi: 10.1136/annrheumdis-2019-216343 31900304 PMC7034352

[30] Tsao CW Aday AW Almarzooq ZI Anderson CAM Arora P Avery CL. Heart disease and stroke statistics-2023 Update: a report from the american heart association. Circulation. (2023) 147:e622. doi: 10.1161/CIR.0000000000001123 36695182 PMC12135016

[31] Bossuyt PM Reitsma JB Bruns DE Gatsonis CA Glasziou PP Irwig L. STARD 2015: an updated list of essential items for reporting diagnostic accuracy studies. BMJ. (2015) 351:h5527. doi: 10.1136/bmj.h5527 26511519 PMC4623764

[32] Visseren FLJ Mach F Smulders YM Carballo D Koskinas KC Bäck M. 2021 ESC guidelines on cardiovascular disease prevention in clinical practice: developed by the task force for cardiovascular disease prevention in clinical practice with representatives of the European Society of Cardiology and 12 medical societies with the special contribution of the European Association of Preventive Cardiology (EAPC). Rev Esp Cardiol (Engl Ed). (2022) 75:429. doi: 10.1016/j.rec.2022.04.003 35525570

[33] Dejaco C Ramiro S Duftner C Besson FL Bley TA Blockmans D. EULAR recommendations for the use of imaging in large vessel vasculitis in clinical practice. Ann Rheum Dis. (2018) 77:636–43. doi: 10.1136/annrheumdis-2017-212649 29358285

[34] Moreel L Betrains A Doumen M Molenberghs G Vanderschueren S Blockmans D. Diagnostic yield of combined cranial and large vessel PET/CT, ultrasound and MRI in giant cell arteritis: a systematic review and meta-analysis. Autoimmun Rev. (2023) 22:103355. doi: 10.1016/j.autrev.2023.103355 37146926

[35] Bosch P Bond M Dejaco C Ponte C Mackie SL Falzon L. Imaging in diagnosis, monitoring and outcome prediction of large vessel vasculitis: a systematic literature review and meta-analysis informing the 2023 update of the EULAR recommendations. RMD Open. (2023) 9:e003379. doi: 10.1136/rmdopen-2023-003379 37620113 PMC10450079

[36] Prearo I Dekorsy FJ Brendel M Lottspeich C Dechant C Schulze-Koops H. Diagnostic yield of axillary artery ultrasound in addition to temporal artery ultrasound for the diagnosis of giant cell arteritis. Clin Exp Rheumatol. (2022) 40:819–25. doi: 10.55563/clinexprheumatol/v1bvfz 35522542

[37] Czihal M Köhler A Lottspeich C Prearo I Hoffmann U Schulze-Koops H. Temporal artery compression sonography for the diagnosis of giant cell arteritis in elderly patients with acute ocular arterial occlusions. Rheumatology (Oxford). (2021) 60:2190–6. doi: 10.1093/rheumatology/keaa515 33123722

