# Supplementary material for: Ultrasound intima-media thickness cut-off values for the diagnosis of giant cell arteritis using a dual clinical and MRI reference standard and cardiovascular risk stratification
Source: Front Med (Lausanne). 2024 Apr 9;11:1389655. doi: 10.3389/fmed.2024.1389655 (PMC11037081; doi:10.3389/fmed.2024.1389655)
Supplement: Supplementary file 1 [file Data_Sheet_1.PDF]

Supplementary Material

Supplementary Figure S1. Patient flow. GCA, giant cell arteritis; MRI, magnetic resonance imaging; US, ultrasound.

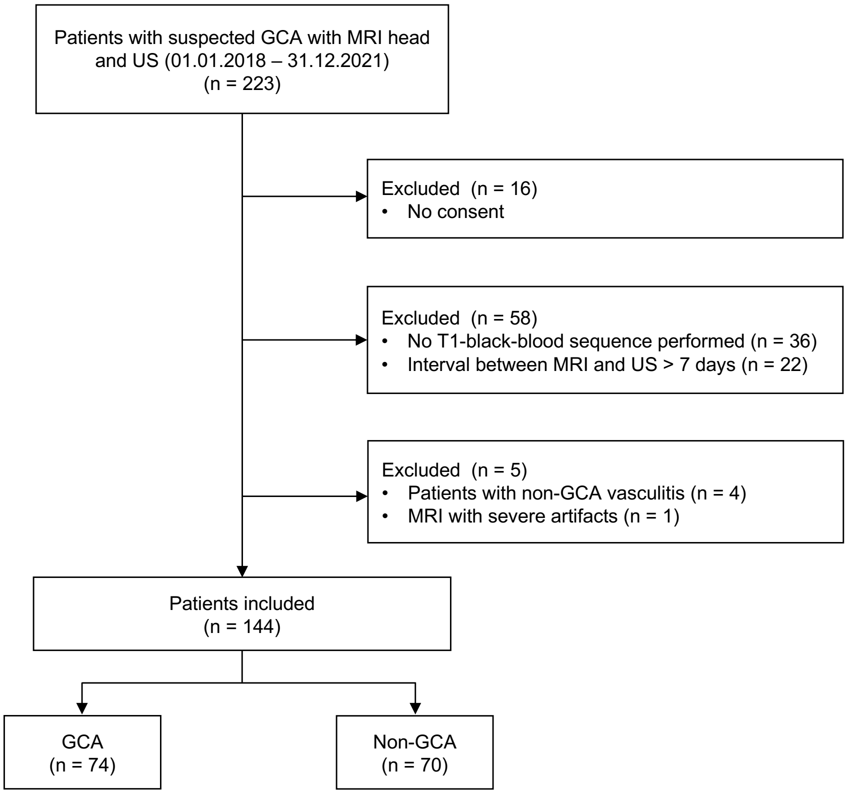

Supplementary Table S1. Clinical diagnosis of 70 non-GCA patients

Expert Diagnosis at 6 months	n (%)
Polymyalgia rheumatica	23 (32.9%)
Headache (not due to GCA)	10 (14.3%)
Polyarthritis	10 (14.3%)
Anterior ischemic optic neuropathy - non-arteritic form	9 (12.8%)
Sarcoidosis; Infection; Lymphoma; Normal temporal artery; Occlusion of retinal artery (not due to GCA). [a]	10 (14.3%)
Abducens palsy; Atherosclerosis; Calcium pyrophosphate deposition disease; Hypertensive urgency; Ischemic stroke; Organizing pneumonia; Spondyloarthritis; Systemic inflammation of unknown aetiology. [b]	8 (11.4%)

[a], Two patients each; [b] One patient each. GCA, giant cell arteritis.

Supplementary Figure S2. Positive and negative likelihood ratios per segment and overall for different IMT cut-offs. N indicates the number of segments (maximum of two per patient, per segment). The optimal cut-off is indicated as a red circle. Shaded areas show 95% confidence regions. CSTA, common superficial temporal artery; IMT, intima-media thickness; TA, temporal artery.

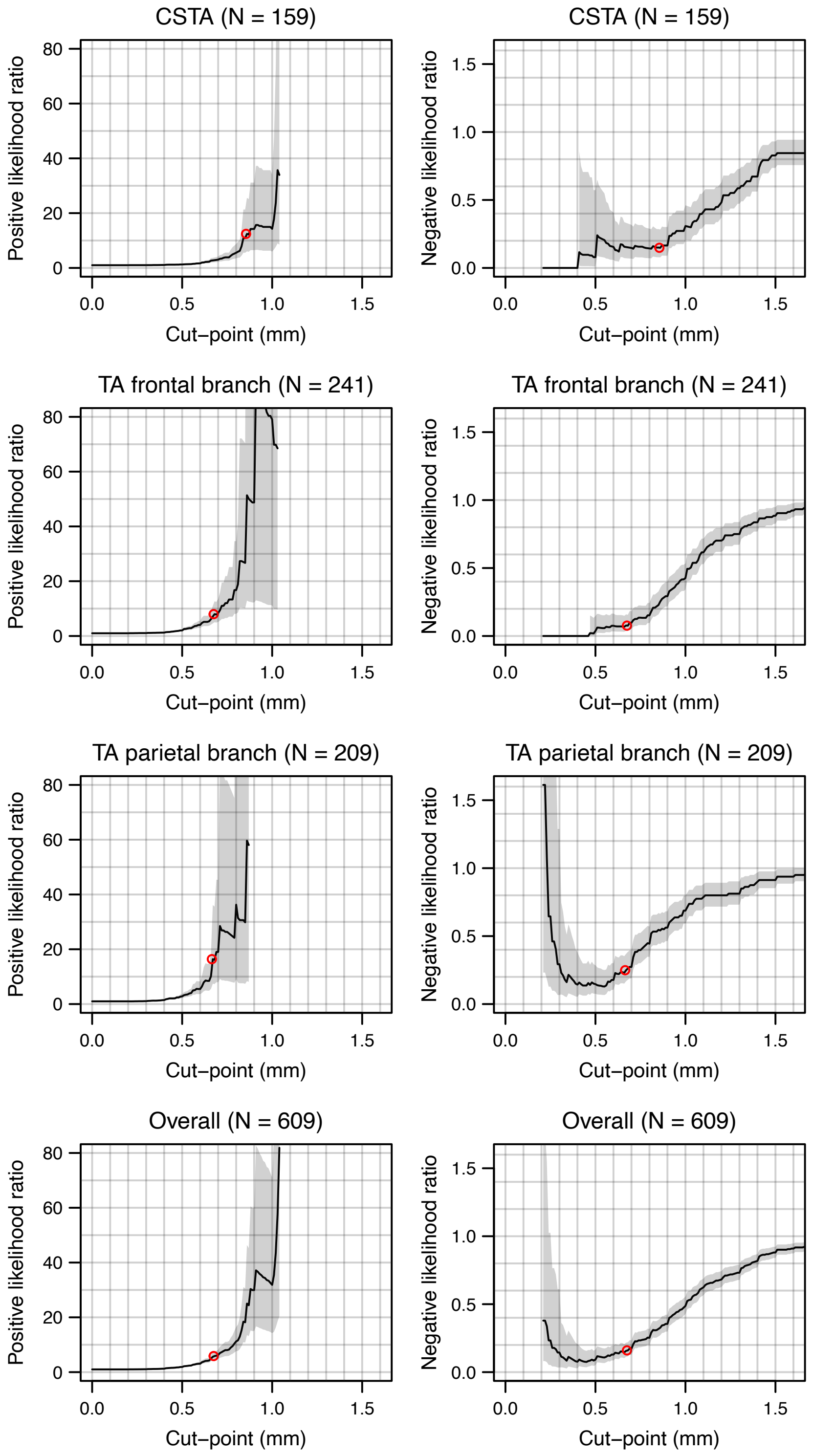

Supplementary Table S2. Sensitivity, specificity, positive and negative likelihood ratios by segment at different cut-offs. CI, confidence interval; GCA, giant cell arteritis.

Cut-off (mm)	≥ cut-off / GCA	Sensitivity (95% CI)	< cut-off / no GCA	Specificity (95% CI)	Positive likelihood ratio (95% CI)	Negative likelihood ratio (95% CI)
**Common superficial temporal artery**						
0	58/58	100.0% (93.8 to 100.0%)	0/101	0.0% (0.0 to 3.7%)		
0.1	58/58	100.0% (93.8 to 100.0%)	0/101	0.0% (0.0 to 3.7%)		
0.2	58/58	100.0% (93.8 to 100.0%)	0/101	0.0% (0.0 to 3.7%)		
0.3	58/58	100.0% (93.8 to 100.0%)	2/101	2.0% (0.5 to 6.9%)	1.02 (0.99 to 1.05)	
0.4	58/58	100.0% (93.8 to 100.0%)	8/101	7.9% (4.1 to 14.9%)	1.09 (1.03 to 1.15)	
0.5	57/58	98.3% (90.9 to 99.7%)	22/101	21.8% (14.8 to 30.8%)	1.26 (1.13 to 1.40)	0.08 (0.01 to 0.57)
0.6	54/58	93.1% (83.6 to 97.3%)	45/101	44.6% (35.2 to 54.3%)	1.68 (1.39 to 2.03)	0.15 (0.06 to 0.41)
0.7	52/58	89.7% (79.2 to 95.2%)	71/101	70.3% (60.8 to 78.3%)	3.02 (2.21 to 4.13)	0.15 (0.07 to 0.32)
0.8	51/58	87.9% (77.1 to 94.0%)	85/101	84.2% (75.8 to 90.0%)	5.55 (3.51 to 8.79)	0.14 (0.07 to 0.29)
0.9	49/58	84.5% (73.1 to 91.6%)	95/101	94.1% (87.6 to 97.2%)	14.22 (6.49 to 31.14)	0.16 (0.09 to 0.30)
1.0	41/58	70.7% (58.0 to 80.8%)	96/101	95.0% (88.9 to 97.9%)	14.28 (5.98 to 34.10)	0.31 (0.21 to 0.46)
1.1	34/58	58.6% (45.8 to 70.4%)	101/101	100.0% (96.3 to 100.0%)		0.41 (0.30 to 0.56)
1.2	30/58	51.7% (39.2 to 64.1%)	101/101	100.0% (96.3 to 100.0%)		0.48 (0.37 to 0.63)
1.3	24/58	41.4% (29.6 to 54.2%)	101/101	100.0% (96.3 to 100.0%)		0.59 (0.47 to 0.73)
1.4	19/58	32.8% (22.1 to 45.6%)	101/101	100.0% (96.3 to 100.0%)		0.67 (0.56 to 0.80)
1.5	10/58	17.2% (9.6 to 28.9%)	101/101	100.0% (96.3 to 100.0%)		0.83 (0.74 to 0.93)
1.6	9/58	15.5% (8.4 to 26.9%)	101/101	100.0% (96.3 to 100.0%)		0.84 (0.76 to 0.94)
1.7	9/58	15.5% (8.4 to 26.9%)	101/101	100.0% (96.3 to 100.0%)		0.84 (0.76 to 0.94)
1.8	6/58	10.3% (4.8 to 20.8%)	101/101	100.0% (96.3 to 100.0%)		0.90 (0.82 to 0.98)
1.9	2/58	3.4% (1.0 to 11.7%)	101/101	100.0% (96.3 to 100.0%)		0.97 (0.92 to 1.01)
**Temporal artery - frontal branch**						
0	104/104	100.0% (96.4 to 100.0%)	0/137	0.0% (0.0 to 2.7%)		
0.1	104/104	100.0% (96.4 to 100.0%)	0/137	0.0% (0.0 to 2.7%)		
0.2	104/104	100.0% (96.4 to 100.0%)	0/137	0.0% (0.0 to 2.7%)		
0.3	104/104	100.0% (96.4 to 100.0%)	8/137	5.8% (3.0 to 11.1%)	1.06 (1.02 to 1.11)	
0.4	104/104	100.0% (96.4 to 100.0%)	26/137	19.0% (13.3 to 26.4%)	1.23 (1.14 to 1.34)	
0.5	102/104	98.1% (93.3 to 99.5%)	71/137	51.8% (43.5 to 60.0%)	2.04 (1.71 to 2.43)	0.04 (0.01 to 0.15)
0.6	98/104	94.2% (88.0 to 97.3%)	105/137	76.6% (68.9 to 82.9%)	4.03 (2.97 to 5.48)	0.08 (0.03 to 0.16)
0.7	95/104	91.3% (84.4 to 95.4%)	122/137	89.1% (82.7 to 93.3%)	8.34 (5.16 to 13.50)	0.10 (0.05 to 0.18)
0.8	89/104	85.6% (77.6 to 91.1%)	130/137	94.9% (89.8 to 97.5%)	16.75 (8.10 to 34.61)	0.15 (0.10 to 0.24)
0.9	74/104	71.2% (61.8 to 79.0%)	135/137	98.5% (94.8 to 99.6%)	48.74 (12.25 to 193.97)	0.29 (0.22 to 0.40)
1.0	60/104	57.7% (48.1 to 66.7%)	136/137	99.3% (96.0 to 99.9%)	79.04 (11.14 to 560.96)	0.43 (0.34 to 0.53)
1.1	40/104	38.5% (29.7 to 48.1%)	137/137	100.0% (97.3 to 100.0%)		0.62 (0.53 to 0.72)
1.2	31/104	29.8% (21.9 to 39.2%)	137/137	100.0% (97.3 to 100.0%)		0.70 (0.62 to 0.80)
1.3	26/104	25.0% (17.7 to 34.1%)	137/137	100.0% (97.3 to 100.0%)		0.75 (0.67 to 0.84)
1.4	17/104	16.3% (10.5 to 24.6%)	137/137	100.0% (97.3 to 100.0%)		0.84 (0.77 to 0.91)
1.5	12/104	11.5% (6.7 to 19.1%)	137/137	100.0% (97.3 to 100.0%)		0.88 (0.83 to 0.95)
1.6	8/104	7.7% (3.9 to 14.4%)	137/137	100.0% (97.3 to 100.0%)		0.92 (0.87 to 0.98)
1.7	6/104	5.8% (2.7 to 12.0%)	137/137	100.0% (97.3 to 100.0%)		0.94 (0.90 to 0.99)
1.8	4/104	3.8% (1.5 to 9.5%)	137/137	100.0% (97.3 to 100.0%)		0.96 (0.93 to 1.00)
**Temporal artery - parietal branch**						
0	80/80	100.0% (95.4 to 100.0%)	0/129	0.0% (0.0 to 2.9%)		
0.1	80/80	100.0% (95.4 to 100.0%)	0/129	0.0% (0.0 to 2.9%)		
0.2	80/80	100.0% (95.4 to 100.0%)	0/129	0.0% (0.0 to 2.9%)		
0.3	78/80	97.5% (91.3 to 99.3%)	11/129	8.5% (4.8 to 14.6%)	1.07 (1.00 to 1.14)	0.29 (0.07 to 1.29)
0.4	76/80	95.0% (87.8 to 98.0%)	45/129	34.9% (27.2 to 43.4%)	1.46 (1.27 to 1.67)	0.14 (0.05 to 0.38)
0.5	72/80	90.0% (81.5 to 94.8%)	88/129	68.2% (59.8 to 75.6%)	2.83 (2.18 to 3.68)	0.15 (0.08 to 0.29)
0.6	68/80	85.0% (75.6 to 91.2%)	109/129	84.5% (77.3 to 89.7%)	5.48 (3.63 to 8.29)	0.18 (0.10 to 0.30)
0.7	59/80	73.8% (63.2 to 82.1%)	124/129	96.1% (91.2 to 98.3%)	19.03 (7.98 to 45.38)	0.27 (0.19 to 0.39)
0.8	45/80	56.2% (45.3 to 66.6%)	127/129	98.4% (94.5 to 99.6%)	36.28 (9.05 to 145.46)	0.44 (0.35 to 0.57)
0.9	35/80	43.8% (33.4 to 54.7%)	129/129	100.0% (97.1 to 100.0%)		0.56 (0.46 to 0.68)
1.0	25/80	31.2% (22.2 to 42.1%)	129/129	100.0% (97.1 to 100.0%)		0.69 (0.59 to 0.80)
1.1	17/80	21.2% (13.7 to 31.4%)	129/129	100.0% (97.1 to 100.0%)		0.79 (0.70 to 0.88)
1.2	16/80	20.0% (12.7 to 30.0%)	129/129	100.0% (97.1 to 100.0%)		0.80 (0.72 to 0.89)
1.3	15/80	18.8% (11.7 to 28.7%)	129/129	100.0% (97.1 to 100.0%)		0.81 (0.73 to 0.90)
1.4	8/80	10.0% (5.2 to 18.5%)	129/129	100.0% (97.1 to 100.0%)		0.90 (0.84 to 0.97)
1.5	7/80	8.8% (4.3 to 17.0%)	129/129	100.0% (97.1 to 100.0%)		0.91 (0.85 to 0.98)
1.6	5/80	6.2% (2.7 to 13.8%)	129/129	100.0% (97.1 to 100.0%)		0.94 (0.89 to 0.99)
1.7	3/80	3.8% (1.3 to 10.5%)	129/129	100.0% (97.1 to 100.0%)		0.96 (0.92 to 1.01)

(continued on next page)

Table S2 (continued)

Cut-off (mm)	≥ cut-off / GCA	Sensitivity (95%-CI)	< cut-off / no GCA	Specificity (95% CI)	Positive likelihood ratio (95% CI)	Negative likelihood ratio (95% CI)
**Overall – All segments**						
0	242/242	100.0% (98.4 to 100.0%)	0/367	0.0% (0.0 to 1.0%)		
0.1	242/242	100.0% (98.4 to 100.0%)	0/367	0.0% (0.0 to 1.0%)		
0.2	242/242	100.0% (98.4 to 100.0%)	0/367	0.0% (0.0 to 1.0%)		
0.3	240/242	99.2% (97.0 to 99.8%)	21/367	5.7% (3.8 to 8.6%)	1.05 (1.02 to 1.08)	0.14 (0.03 to 0.61)
0.4	238/242	98.3% (95.8 to 99.4%)	79/367	21.5% (17.6 to 26.0%)	1.25 (1.18 to 1.33)	0.08 (0.03 to 0.21)
0.5	231/242	95.5% (92.0 to 97.4%)	181/367	49.3% (44.2 to 54.4%)	1.88 (1.70 to 2.09)	0.09 (0.05 to 0.17)
0.6	220/242	90.9% (86.6 to 93.9%)	259/367	70.6% (65.7 to 75.0%)	3.09 (2.62 to 3.64)	0.13 (0.09 to 0.19)
0.7	206/242	85.1% (80.1 to 89.1%)	317/367	86.4% (82.5 to 89.5%)	6.25 (4.80 to 8.13)	0.17 (0.13 to 0.23)
0.8	185/242	76.4% (70.7 to 81.4%)	342/367	93.2% (90.1 to 95.3%)	11.22 (7.64 to 16.49)	0.25 (0.20 to 0.32)
0.9	158/242	65.3% (59.1 to 71.0%)	359/367	97.8% (95.8 to 98.9%)	29.95 (15.00 to 59.80)	0.35 (0.30 to 0.42)
1.0	126/242	52.1% (45.8 to 58.3%)	361/367	98.4% (96.5 to 99.2%)	31.85 (14.27 to 71.07)	0.49 (0.43 to 0.56)
1.1	91/242	37.6% (31.7 to 43.9%)	367/367	100.0% (99.0 to 100.0%)		0.62 (0.57 to 0.69)
1.2	77/242	31.8% (26.3 to 37.9%)	367/367	100.0% (99.0 to 100.0%)		0.68 (0.63 to 0.74)
1.3	65/242	26.9% (21.7 to 32.8%)	367/367	100.0% (99.0 to 100.0%)		0.73 (0.68 to 0.79)
1.4	44/242	18.2% (13.8 to 23.5%)	367/367	100.0% (99.0 to 100.0%)		0.82 (0.77 to 0.87)
1.5	29/242	12.0% (8.5 to 16.7%)	367/367	100.0% (99.0 to 100.0%)		0.88 (0.84 to 0.92)
1.6	22/242	9.1% (6.1 to 13.4%)	367/367	100.0% (99.0 to 100.0%)		0.91 (0.87 to 0.95)
1.7	18/242	7.4% (4.8 to 11.4%)	367/367	100.0% (99.0 to 100.0%)		0.93 (0.89 to 0.96)
1.8	10/242	4.1% (2.3 to 7.4%)	367/367	100.0% (99.0 to 100.0%)		0.96 (0.93 to 0.98)
1.9	2/242	0.8% (0.2 to 3.0%)	367/367	100.0% (99.0 to 100.0%)		0.99 (0.98 to 1.00)

Supplementary Table S3. Patient-level measures of diagnostic accuracy for statistically best cut-offs and range of possible cut-offs with minimum specificities per segment of 85 to 100%. GCA, giant cell arteritis; CI, confidence interval; CVR, cardiovascular risk; N, number of patients in the subpopulation.

	**Total study population (N = 144)**						**Patients with cranial manifestations (N = 117)**						**Patients without high/very high CVR (N = 90)**						**Patients with high/very high CVR (N = 54)**					
	**Abnormal / GCA**	**Sensitivity (95% CI)**	**Normal / no GCA**	**Specificity (95% CI)**	**n/N**	**Correctly classified (95% CI)**	**Abnormal / GCA**	**Sensitivity (95% CI)**	**Normal / no GCA**	**Specificity (95% CI)**	**n/N**	**Correctly classified (95% CI)**	**Abnormal / GCA**	**Sensitivity (95% CI)**	**Normal / no GCA**	**Specificity (95% CI)**	**n/N**	**Correctly classified (95% CI)**	**Abnormal / GCA**	**Sensitivity (95% CI)**	**Normal / no GCA**	**Specificity (95% CI)**	**n/N**	**Correctly classified (95% CI)**
**Statistically optimal cut-offs**	64 / 74	86.5% (76.9 – 92.5%)	57 / 70	81.4% (70.8 – 88.8%)	121/ 144	84.0% (77.2 – 89.1%)	58 / 63	92.1% (82.7 – 96.6%)	47 / 54	87% (75.6 – 93.6%)	105/ 117	89.7% (82.9 – 94.0%)	43 / 48	89.6% (77.8 – 95.5%)	38 / 42	90.5% (77.9 – 96.2%)	81/ 90	90.0% (82.1 – 94.5%)	21 / 26	80.8% (62.1 – 91.5%)	19 / 28	67.9% (49.3 – 82.1%	40/ 54	74.1% (61.1 – 83.9%)
**Specificity 85%**	64 / 74	86.5% (76.9 – 92.5%)	53 / 70	75.7% (64.5 – 84.2%)	117/ 144	81.2% (74.1 – 86.8%)	58 / 63	92.1% (82.7 – 96.6%)	43 / 54	79.6% (67.1 – 88.2%)	101/ 117	86.3% (78.9 – 91.4%)	43 / 48	89.6% (77.8 – 95.5%)	36 / 42	85.7% (72.2 – 93.3%)	79/ 90	87.8% (79.4 – 93.0%)	21 / 26	80.8% (62.1 – 91.5%)	17 / 28	60.7% (42.4 – 76.4%)	38/ 54	70.4% (57.2 – 80.9%)
**Specificity 86%**	64 / 74	86.5% (76.9 – 92.5%)	53 / 70	75.7% (64.5 – 84.2%)	117/ 144	81.2% (74.1 – 86.8%)	58 / 63	92.1% (82.7 – 96.6%)	43 / 54	79.6% (67.1 – 88.2%)	101/ 117	86.3% (78.9 – 91.4%)	43 / 48	89.6% (77.8 – 95.5%)	36 / 42	85.7% (72.2 – 93.3%)	79/ 90	87.8% (79.4 – 93.0%)	21 / 26	80.8% (62.1 – 91.5%)	17 / 28	60.7% (42.4 – 76.4%)	38/ 54	70.4% (57.2 – 80.9%)
**Specificity 87%**	64 / 74	86.5% (76.9 – 92.5%)	54 / 70	77.1% (66.0 – 85.4%)	118/ 144	81.9% (74.9 – 87.4%)	58 / 63	92.1% (82.7 – 96.6%)	44 / 54	81.5% (69.2 – 89.6%)	102/ 117	87.2% (79.9 – 92.1%)	43 / 48	89.6% (77.8 – 95.5%)	37 / 42	88.1% (75.0 – 94.8%)	80/ 90	88.9% (80.7 – 93.9%)	21 / 26	80.8% (62.1 – 91.5%)	17 / 28	60.7% (42.4 – 76.4%)	38/ 54	70.4% (57.2 – 80.9%)
**Specificity 88%**	64 / 74	86.5% (76.9 – 92.5%)	54 / 70	77.1% (66.0 – 85.4%)	118/ 144	81.9% (74.9 – 87.4%)	58 / 63	92.1% (82.7 – 96.6%)	44 / 54	81.5% (69.2 – 89.6%)	102/ 117	87.2% (79.9 – 92.1%)	43 / 48	89.6% (77.8 – 95.5%)	37 / 42	88.1% (75.0 – 94.8%)	80/ 90	88.9% (80.7 – 93.9%)	21 / 26	80.8% (62.1 – 91.5%)	17 / 28	60.7% (42.4 – 76.4%)	38/ 54	70.4% (57.2 – 80.9%)
**Specificity 89%**	64 / 74	86.5% (76.9 – 92.5%)	54 / 70	77.1% (66.0 – 85.4%)	118/ 144	81.9% (74.9 – 87.4%)	58 / 63	92.1% (82.7 – 96.6%)	44 / 54	81.5% (69.2 – 89.6%)	102/ 117	87.2% (79.9 – 92.1%)	43 / 48	89.6% (77.8 – 95.5%)	37 / 42	88.1% (75.0 – 94.8%)	80/ 90	88.9% (80.7 – 93.9%)	21 / 26	80.8% (62.1 – 91.5%)	17 / 28	60.7% (42.4 – 76.4%)	38/ 54	70.4% (57.2 – 80.9%)
**Specificity 90%**	64 / 74	86.5% (76.9 – 92.5%)	55 / 70	78.6% (67.6 – 86.6%)	119/ 144	82.6% (75.6 – 88.0%)	58 / 63	92.1% (82.7 – 96.6%)	44 / 54	81.5% (69.2 – 89.6%)	102/ 117	87.2% (79.9 – 92.1%)	43 / 48	89.6% (77.8 – 95.5%)	38 / 42	90.5% (77.9 – 96.2%)	81/ 90	90.0% (82.1 – 94.6%)	21 / 26	80.8% (62.1 – 91.5%)	17 / 28	60.7% (42.4 – 76.4%)	38/ 54	70.4% (57.2 – 80.9%)
**Specificity 91%**	64 / 74	86.5% (76.9 – 92.5%)	56 / 70	80.0% (69.2 – 87.7%)	120/ 144	83.3% (76.4 – 88.5%)	58 / 63	92.1% (82.7 – 96.6%)	45 / 54	83.3% (71.3 – 91.0%)	103/ 117	88.0% (80.9 – 92.7%)	43 / 48	89.6% (77.8 – 95.5%)	39 / 42	92.9% (81.0 – 97.5%)	82/ 90	91.1% (83.4 – 95.4%)	21 / 26	80.8% (62.1 – 91.5%)	17 / 28	60.7% (42.4 – 76.4%)	38/ 54	70.4% (57.2 – 80.9%)
**Specificity 92%**	63 / 74	85.1% (75.3 – 91.5%)	56 / 70	80.0% (69.2 – 87.7%)	119/ 144	82.6% (75.6 – 88.0%)	58 / 63	92.1% (82.7 – 96.6%)	45 / 54	83.3% (71.3 – 91.0%)	103/ 117	88.0% (80.9 – 92.7%)	42 / 48	87.5% (75.3 – 94.1%)	39 / 42	92.9% (81.0 – 97.5%)	81/ 90	90.0% (82.1 – 94.6%)	21 / 26	80.8% (62.1 – 91.5%)	17 / 28	60.7% (42.4 – 76.4%)	38/ 54	70.4% (57.2 – 80.9%)
**Specificity 93%**	63 / 74	85.1% (75.3 – 91.5%)	60 / 70	85.7% (75.7 – 92.1%)	123/ 144	85.4% (78.7 – 90.3%)	58 / 63	92.1% (82.7 – 96.6%)	49 / 54	90.7% (80.1 – 96.0%)	107/ 117	91.5% (85.0 – 95.3%)	42 / 48	87.5% (75.3 – 94.1%)	40 / 42	95.2% (84.2 – 98.7%)	82/ 90	91.1% (83.4 – 95.4%)	21 / 26	80.8% (62.1 – 91.5%)	20 / 28	71.4% (52.9 – 84.7%)	41/ 54	75.9% (63.1 – 85.4%)
**Specificity 94%**	62 / 74	83.8% (73.8 – 90.5%)	60 / 70	85.7% (75.7 – 92.1%)	122/ 144	84.7% (78.0 – 89.7%)	58 / 63	92.1% (82.7 – 96.6%)	49 / 54	90.7% (80.1 – 96.0%)	107/ 117	91.5% (85.0 – 95.3%)	42 / 48	87.5% (75.3 – 94.1%)	40 / 42	95.2% (84.2 – 98.7%)	82/ 90	91.1% (83.4 – 95.4%)	20 / 26	76.9% (57.9 – 89.0%)	20 / 28	71.4% (52.9 – 84.7%)	40/ 54	74.1% (61.1 – 83.9%)
**Specificity 95%**	60 / 74	81.1% (70.7 – 88.4%)	60 / 70	85.7% (75.7 – 92.1%)	120/ 144	83.3% (76.4 – 88.5%)	56 / 63	88.9% (78.8 – 94.5%)	49 / 54	90.7% (80.1 – 96.0%)	105/ 117	89.7% (82.9 – 94.0%)	40 / 48	83.3% (70.4 – 91.3%)	40 / 42	95.2% (84.2 – 98.7%)	80/ 90	88.9% (80.7 – 93.9%)	20 / 26	76.9% (57.9 – 89.0%)	20 / 28	71.4% (52.9 – 84.7%)	40/ 54	74.1% (61.1 – 83.9%)
**Specificity 96%**	59 / 74	79.7% (69.2 – 87.3%)	63 / 70	90.0% (80.8 – 95.1%)	122/ 144	84.7% (78.0 – 89.7%)	55 / 63	87.3% (76.9 – 93.4%)	51 / 54	94.4% (84.9 – 98.1%)	106/ 117	90.6% (83.9 – 94.7%)	40 / 48	83.3% (70.4 – 91.3%)	41 / 42	97.6% (87.7 – 99.6%)	81/ 90	90.0% (82.1 – 94.6%)	19 / 26	73.1% (53.9 – 86.3%)	22 / 28	78.6% (60.5 – 89.8%)	41/ 54	75.9% (63.1 – 85.4%)
**Specificity 97%**	57 / 74	77.0% (66.3 – 85.1%)	64 / 70	91.4% (82.5 – 96.0%)	121/ 144	84.0% (77.2 – 89.1%)	53 / 63	84.1% (73.2 – 91.1%)	51 / 54	94.4% (84.9 – 98.1%)	104/ 117	88.9% (81.9 – 93.4%)	38 / 48	79.2% (65.7 – 88.3%)	41 / 42	97.6% (87.7 – 99.6%)	79/ 90	87.8% (79.4 – 93.0%)	19 / 26	73.1% (53.9 – 86.3%)	23 / 28	82.1% (64.4 – 92.1%)	42/ 54	77.8% (65.1 – 86.8%)
**Specificity 98%**	54 / 74	73.0% (61.9 – 81.8%)	66 / 70	94.3% (86.2 – 97.8%)	120/ 144	83.3% (76.4 – 88.5%)	50 / 63	79.4% (67.8 – 87.5%)	52 / 54	96.3% (87.5 – 99.0%)	102/ 117	87.2% (79.9 – 92.1%)	35 / 48	72.9% (59.0 – 83.4%)	41 / 42	97.6% (87.7 – 99.6%)	76/ 90	84.4% (75.6 – 90.5%)	19 / 26	73.1% (53.9 – 86.3%)	25 / 28	89.3% (72.8 – 96.3%)	44/ 54	81.5% (69.2 – 89.6%)
**Specificity 99%**	51 / 74	68.9% (57.7 – 78.3%)	69 / 70	98.6% (92.3 – 99.7%)	120/ 144	83.3% (76.4 – 88.5%)	47 / 63	74.6% (62.7 – 83.7%)	53 / 54	98.1% (90.2 – 99.7%)	100/ 117	85.5% (78.0 – 90.7%)	33 / 48	68.8% (54.7 – 80.1%)	42 / 42	100% (91.6 – 100%)	75/ 90	83.3% (74.3 – 89.6%)	18 / 26	69.2% (50.0 – 83.5%)	27 / 28	96.4% (82.3 – 99.4%)	45/ 54	83.3% (71.3 – 91.0%)
**Specificity 100%**	47 / 74	63.5% (52.1 – 73.6%)	70 / 70	100% (94.8 – 100%)	117/ 144	81.2% (74.1 – 86.8%)	43 / 63	68.3% (56.0 – 78.4%)	54 / 54	100% (93.4 – 100%)	97/ 117	82.9% (75.1 – 88.7%)	30 / 48	62.5% (48.4 – 74.8%)	42 / 42	100% (91.6 – 100%)	72/ 90	80.0% (70.6 – 87.0%)	17 / 26	65.4% (46.2 – 80.6%)	28 / 28	100% (87.9 – 100%)	45/ 54	83.3% (71.3 – 91.0%)
